# Effect of acetic acid bacteria colonization on oviposition and feeding site choice in *Drosophila suzukii* and its related species

**DOI:** 10.17912/micropub.biology.001111

**Published:** 2024-02-06

**Authors:** Airi Sato, Joanne Y. Yew, Aya Takahashi

**Affiliations:** 1 Department of Biological Sciences, Tokyo Metropolitan University, Hachioji, Tokyo, Japan; 2 Pacific Biosciences Research Center, University of Hawaiʻi at Mānoa, Honolulu, Hawaii, United States; 3 Research Center for Genomics and Bioinformatics, Tokyo Metropolitan University, Hachioji, Tokyo, Japan

## Abstract

Unlike many species of *Drosophila* flies that colonize decaying fruits, *Drosophila suzukii* lay eggs in ripening fruits. The oviposition and feeding site preferences for bacterial growth were quantified in multiple strains of *D. suzukii* and its closely related species, *D. subpulchrella* and *D. biarmipes* . A continuous degree of preference for oviposition sites with *Acetobacter* growth both within and across species suggested that the separation in resource usage is notable but not complete among these species. The lack of interspecific differences in feeding site preference for *Acetobacter* -containing media implied that the oviposition site preferences evolved independently from the feeding site preference.

**Figure 1. Oviposition and feeding site preferences for the media containing Acetobacter sp.**
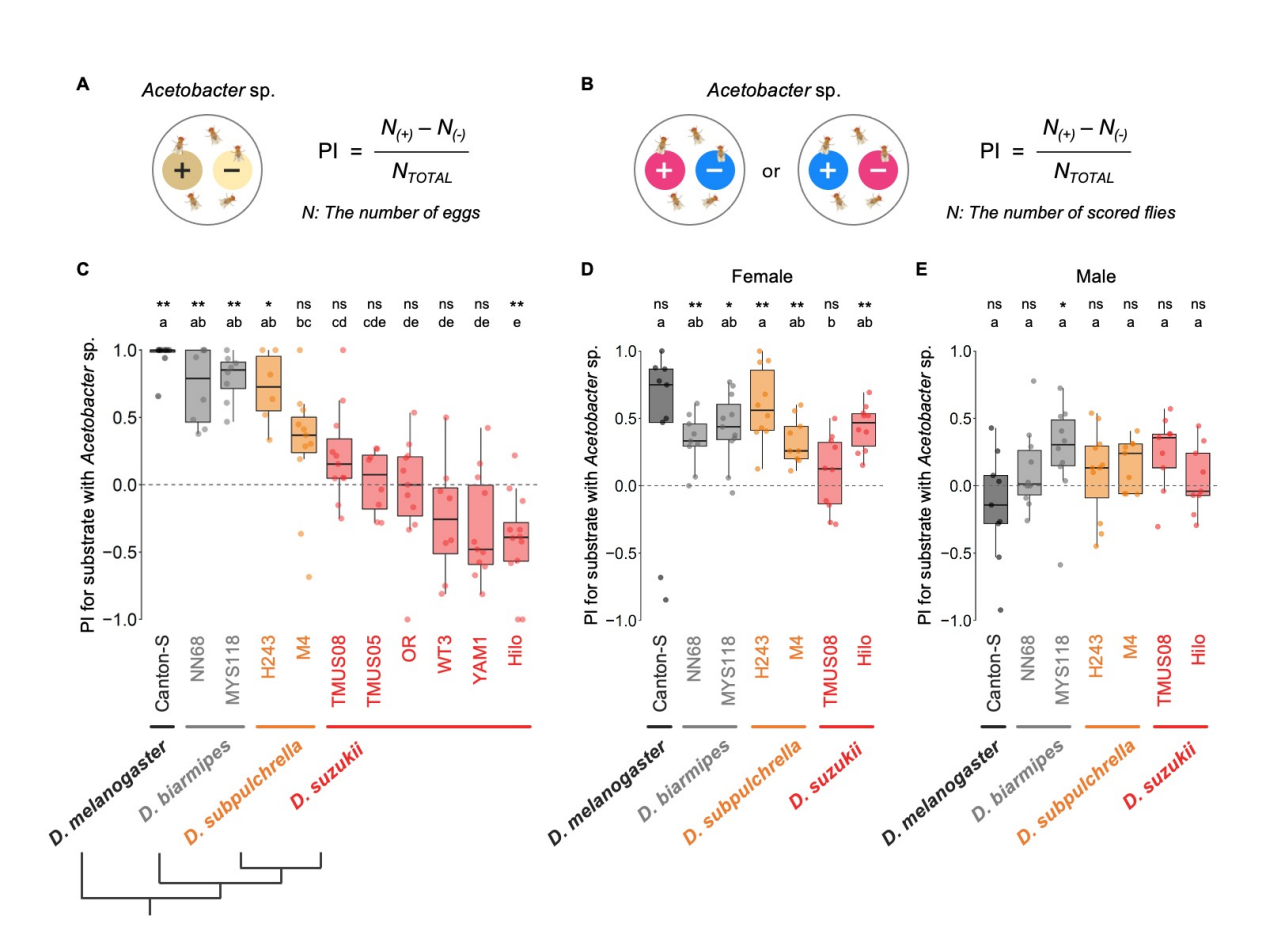
(A) Preference index (PI) calculated for the oviposition assay. + and - indicate the presence and absence of microbial growth on the media, respectively. (B) PI calculated for the feeding assay. + and - indicate the presence and absence of microbial growth on the media, respectively. (C) Oviposition site preference (quantified as PI) of *D. suzukii* and related species, shown with their phylogenetic relationship (topology) (Suvorov et al., 2022). Results from assays with fewer than 15 eggs on either substrate were excluded from the analyses. Replicates are represented by dots (n = 6–12 replicates per strain). (D) Female feeding site preference (quantified as PI) of *D. suzukii* and its related species. (E) Male feeding site preference (quantified as PI) of *D. suzukii* and its related species. For the feeding experiments in (D) and (E), trials where less than 80% of flies or fewer than 20 flies could be scored with dyed abdomens were excluded from the analyses. Replicates are represented by dots (n = 9–11 replicates per strain). For all graphs in (C–E), box signifies the upper and lower quartiles, and horizontal bar indicates median. Upper and lower whiskers represent the maximum and minimum 1.5 × interquartile range, respectively. The results from two types of statistical analysis are shown above the graph; the first row indicates the results from two-sided binominal tests assuming an underlying 1:1 proportion (*: *p* < 0.05, **: *p* < 0.01, ns: *p* ≥ 0.05), and the second row indicates the results from the Kruskal-Wallis tests followed by Dunn’s tests with Benjamin-Hochberg FDR correction ( *p* < 0.05).

## Description

Fermenting fruits are a nutrient-rich food resource for many insects, providing a diet rich with yeasts, bacteria, and an abundant supply of proteins (Begon 1982) . The majority of *Drosophila* species lay eggs on fermenting or rotting fruits. However, the females of *Drosophila suzukii* , the spotted wing drosophila, are known to lay eggs into ripening fruits with a relatively low protein-to-carbohydrate ratio (P:C) by using an enlarged and serrated ovipositor (oviscapt or hypogynium) (Walsh et al., 2011; Cini et al., 2012; Atallah et al., 2014; Karageorgi et al., 2017; Muto et al., 2018) . This behavior, which causes significant agricultural damage in the invaded areas (Cini et al., 2012; Asplen et al., 2015) , has allowed the offspring to utilize the host fruit earlier and avoid competition (Kienzle et al., 2020) .

However, considering that *D. suzukii* larvae have limited physiological adaptation to a low-protein diet and intact healthy fruits have seasonally restricted availability, the competitive advantage of ovipositing in ripening fruits can be conditional (Silva-Soares et al., 2017; Young et al., 2018; Kienzle et al., 2020; Deans and Hutchingson 2021) . Therefore, there is likely to be variability in oviposition preference maintained within the species. Also, since adult flies, especially females, require a large amount of protein for reproduction (Jensen et al., 2015) , their foraging decisions will be affected by their own nutritional demands as well (Lihoreau et al., 2016) . Given the potential conflict between nutritional demand and competition for resources, we investigated the following: 1) the degree of interspecific differences and intraspecific variation in preference for oviposition sites that contain microbial species associated with decaying fruits, and 2) whether oviposition site preferences are independent of feeding site selection.

In a previous study by our group, we show that in contrast to the females of *D. melanogaster* , a typical fermenting fruit consumer, the females of *D. suzukii* did not prefer to lay eggs on substrates inoculated with a mixture of microbial species collected from other adult flies (Sato et al., 2021) . In this study, we tested the oviposition preference for a single species of *Acetobacter,* a genus of acetic acid bacteria and a common constituent of the *Drosophila* gut microbiome (Broderick and Lemaitre 2012; Chandler et al., 2014; Vacchini et al., 2017) . The oviposition site preferences for substrates with and without microbial growth were quantified in six strains of *D. suzukii* , two strains from *D. subpulchrella* , which has recently diverged from *D. suzukii* , two strains from *D. biarmipes* , which is the most closely related species examined that prefer oviposition substrate colonized by microbes, and a *D. melanogaster* strain (Keesey et al., 2015; Sato et al., 2021) .

As expected, *D. melanogaster* strongly preferred to lay eggs on the substrate with bacterial growth ( Figure 1A, C ). Similarly, two tested strains of *D. biarmipes* showed strong preferences for *Acetobacter* . In contrast, all the strains of *D. suzukii* showed significantly weaker preference compared to the strains of *D. melanogaster* and *D. biarmipes* , suggesting that the preference for *Acetobacter* in *D. suzukii* is distinct from that in *D. melanogaster* and *D. biarmipes* . However, while the Hilo strain of *D. suzukii* avoided *Acetobacter* when choosing the oviposition site, 5 other strains (TMUS05, TMUS08, OR, WT3 and YAM1) did not show any preference or avoidance ( Figure 1C ). This result implied an intraspecific variation in oviposition site preference for *Acetobacter* in *D. suzukii* .

Regarding *D. subpulchrella* , there was no significant difference in the preference index (PI) between *D. subpulchrella* H243 strain and the strains of *D. melanogaster* and *D. biarmipes* . However, the PI of *D. subpulchrella* M4 strain was significantly different from the strains of *D. melanogaster* and *D. biarmipes* , and exhibited a similar PI to two of the *D. suzukii* strains (TMUS05 and TMUS08). Therefore, this species has an intermediate degree of preference between *D. suzukii* and *D. melanogaster* / *D. biarmipes* , and harbors variation within species. This species also has enlarged and serrated ovipositors (Atallah et al., 2014; Muto et al., 2018) ; however, their tendency to lay eggs into firm ripening fruits is weaker than that of *D. suzukii* (Atallah et al., 2014; Durkin et al., 2021) . The distribution of *D. suzukii* and *D. subpulchrella* is overlapping and can be found sympatrically in many localities in Japan (Sasaki and Abe 1993; Takamori et al., 2006; Mitsui et al., 2010) . Together with previous studies showing intermediate oviposition characteristics of *D. subpulchrella* between *D. melanogaster* and *D. suzukii* (Atallah et al., 2014; Durkin et al., 2021) , our results suggest that the niche separation regarding the oviposition sites between *D. suzukii* and *D. subpulchrella* is not complete.

We also found in this study that in contrast to the oviposition preference, there was no clear interspecific divergence in the feeding preference for media inoculated with *Acetobacter* sp. For females of all the tested strains, the median values of the feeding site PIs for *Acetobacter* were positive, ranging from 0.13 in *D. suzukii* TMUS08 to 0.75 in *D. melanogaster* Canton-S ( Figure 1B, D ). It should be noted that the non-significant binomial test in *D. melanogaster* Canton-S females is likely due to the two lowest PI data points. No fixed differences between species were detected, and in contrast to the oviposition assay, there was no sign of interspecific divergence among these species. For males, all the tested strains showed no-preference except *D. biarmipes* MYS118, and no significant difference in PI was detected between the strains ( Figure 1B, E ). The contrasting result between the oviposition and feeding assay suggests that the interspecific differences in oviposition site preference have different molecular bases and are likely to have evolved independently from the relatively conserved feeding preferences among the tested species.

## Methods

**Fly strains**

The following strains were used: *D. suzukii* strain TMUS05 and TMUS08 collected in Hachioji, Japan, in 2015, *D. suzukii* strain Hilo collected in Hilo, Island of Hawai‘i, U. S. A., in 2017, *D. suzukii* strain OR collected in Oregon, U. S. A., in 2017, *D. suzukii* strain WT3 collected in California, U. S. A., in 2009 and sib-mated for ten generations (Chiu et al., 2013) , *D. suzukii* strain YAM1 collected in Yamagata prefecture, Japan, in 2004, *D. subpulchrella* strain H243 collected in Hiratsuka, Japan, in 1979, *D. subpulchrella* strain M4 collected in Matsumoto, Japan, in 1982, *D. biarmipes* strain MYS118 collected in Mysore, India, in 1981, *D. biarmipes* strain NN68 collected in Nakhonn Nayok, Thailand, in 1977, and *D. melanogaster* strain Canton-S BL#9515. *D. suzukii* and *D. subpulchrella* were maintained at 20 ± 1°C and other strains were maintained at 25 ± 1°C. All the strains were reared under a photoperiod of 12 h. Flies were fed with the standard corn meal food (ingredients per liter of water: 90 g corn meal, 40 g dry yeast, 100 g glucose, 8 g agar, 3 ml propionic acid, 10 ml butyl 4-hydroxybenzoate). *D. suzukii* and *D. subpulchrella* flies aged 10–15 days after eclosion and *D. biarmipes* and *D. melanogaster* flies aged 4–7 days after eclosion were used for the assays.

**Acetic acid bacteria**

Single colonies of acetic acid bacteria were isolated from the microbes collected from the surface of fly-inoculated media and subjected to 16S-rRNA gene sequencing (Sato et al. *,* 2021). The colonies of *Acetobacter* sp. were identified by the 16S-rRNA gene sequences and were maintained in the MRS media at 25–30°C until use.

**Oviposition assay to assess the preference for substrates with *Acetobacter* sp.**

The oviposition assay was conducted in a petri dish (90 mm diameter × 20 mm height, SH90-20, IWAKI) with test and control substrates. The substrates were made from 50% apple juice (SUNPACK, JAN code: 4571247510950), including 1% agar (Drosophila agar type II, Apex), and put in a petri dish (40 mm diameter × 13 mm height). Twenty µL of the bacterial solution (OD = 1 in distilled water) or the control distilled water were spread onto the surface of the substrate and incubated for 24 h at 25 ± 1°C.

To account for interspecific differences in the deposited egg numbers per assay, 10 (for *D. suzukii* and *D. subpulchrella* ) or 5 (for *D. melanogaster* and *D. biarmipes* ) females were placed into each chamber without anesthesia by an aspirator within 4 h before the dark cycle and kept for 16 h under a photoperiod of 12 h. The assay was conducted at 20 ± 1°C for *D. suzukii* and *D. subpulchrella* and at 25 ± 1°C for *D. biarmipes* and *D. melanogaster* to control for their optimal temperatures. After the oviposition assay, photo images of each substrate with eggs were taken by a camera (Olympus OM-D E-M10 MarkII) with transmitted light from the bottom. The number of eggs on each substrate was counted using ImageJ v1.53k (Schneider et al., 2012) .

**Feeding assay for *Acetobacter* sp.**

A binary food choice assay was adapted to analyze feeding site preference using two different dyes. The chamber used for the oviposition assay was also used for the feeding assay, with the exception that the agar medium (50% diluted apple juice and 1% agar) which was dyed with either blue (brilliant blue FCF, 0.125 mg/mL) or red (sulforhodamine B, 0.1 mg/mL) dyes. The microbial solution and the water control were also dyed blue or red using the same concentrations as above. The dye colors were randomly switched for each assay.

Twenty-eight to 63 individuals were starved before the assay in a 50 mL centrifuge tube containing two sheets of Kim-wipe soaked with 3 mL distilled water. The length of starvation time was set differently for each tested group: 24 h for the females of *D. suzukii* , *D. subpulchrella* , and *D. melanogaster* , 26 h for the females of *D. biarmipes* , 22 h for the males of *D. suzukii* , *D. subpulchrella* and *D. biarmipes* , 20 h for the males of *D. melanogaster* . The temperature was kept at 20 ± 1°C for *D. suzukii* and *D. subpulchrella* , and 25 ± 1°C for *D. melanogaster* and *D. biarmipes* . Each strain and sex varied in terms of starvation time. The appropriate lengths of time for starvation and feeding assays for each strain and sex were optimized to ensure that the majority of tested flies fed on the media. Trials where less than 80% of flies or fewer than 20 flies could be scored with dyed abdomens were excluded from the analyses.

After starvation, flies were placed into the feeding chamber without anesthesia and left for 120 min (or 90 min for *D. melanogaster* ). Then, the flies were anesthetized by CO _2_ and kept at -20°C until the abdomen color was scored under the stereomicroscope. Individuals with mixed color abdomens were not scored.
